# Experimental and Theoretical Modal Analysis of Full-Sized Wood Composite Panels Supported on Four Nodes

**DOI:** 10.3390/ma10060683

**Published:** 2017-06-21

**Authors:** Cheng Guan, Houjiang Zhang, Xiping Wang, Hu Miao, Lujing Zhou, Fenglu Liu

**Affiliations:** 1 School of Technology, Beijing Forestry University, Beijing 100083, China; cguan6@bjfu.edu.cn (C.G.); Mh1993@buaa.edu.cn (H.M.); lujingzhou6@gmail.com (L.Z.); liufenglu39@bjfu.edu.cn (F.L.); 2 USDA Forest Service, Forest Products Laboratory, Madison, WI 53726-2398, USA; xwang@fs.fed.us

**Keywords:** wood composite panel, four-node support, modal testing, modal analysis, modulus of elasticity, sensitivity analysis

## Abstract

Key elastic properties of full-sized wood composite panels (WCPs) must be accurately determined not only for safety, but also serviceability demands. In this study, the modal parameters of full-sized WCPs supported on four nodes were analyzed for determining the modulus of elasticity (*E*) in both major and minor axes, as well as the in-plane shear modulus of panels by using a vibration testing method. The experimental modal analysis was conducted on three full-sized medium-density fiberboard (MDF) and three full-sized particleboard (PB) panels of three different thicknesses (12, 15, and 18 mm). The natural frequencies and mode shapes of the first nine modes of vibration were determined. Results from experimental modal testing were compared with the results of a theoretical modal analysis. A sensitivity analysis was performed to identify the sensitive modes for calculating *E* (major axis: *E_x_* and minor axis: *E_y_*) and the in-plane shear modulus (*G_xy_*) of the panels. Mode shapes of the MDF and PB panels obtained from modal testing are in a good agreement with those from theoretical modal analyses. A strong linear relationship exists between the measured natural frequencies and the calculated frequencies. The frequencies of modes (2, 0), (0, 2), and (2, 1) under the four-node support condition were determined as the characteristic frequencies for calculation of *E_x_*, *E_y_*, and *G_xy_* of full-sized WCPs. The results of this study indicate that the four-node support can be used in free vibration test to determine the elastic properties of full-sized WCPs.

## 1. Introduction

Wood composite panels (WCPs), such as medium-density fibreboard (MDF) and particleboard (PB), are widely used in furniture manufacture, packaging, building construction, musical instruments, and other industrial sectors. Full-sized WCPs with a nominal size of 2440 mm × 1220 mm (length × width) are universal in the production and global market. For full-sized WCPs, mechanical properties, such as the modulus of elasticity (*E*) and in-plane shear modulus, are important performance characteristics that should be taken into consideration during manufacturing and quality control processes. Rapid and accurate determination of these elastic properties in full-sized panels is, therefore, very important for meeting the product specifications and ensuring satisfactory performance under different service conditions. 

The application of non-destructive evaluation (NDE) methods, like transverse vibration or longitudinal stress-wave, for elastic properties determination of WCPs has been studied for many years [1,2,3,4,5,6,7,8,9,10,11,12,13,14,15,16,17,18,19,20,21,22]. Among these studies, property evaluation of small wood composite specimens through the beam-vibrational method has been used with good success [6,8,9,10,11,12,13,16,17,22] and a dynamic cantilever beam apparatus for a variety of elastic properties determination of small wood composite materials was presented by Hunt, Zhou, and Guan [12,13,16,22]. However, NDE of full-sized WCPs through panel-vibrational method was still in the research stage. A fundamental aspect of this method is to support a panel in a well-defined position, induce a vibration to the panel by a short impact, and to measure the natural frequencies. Knowing the natural frequencies of different vibration modes and the boundary conditions, the elastic properties of the panel can be calculated, including the *E* in both major and minor axes and the in-plane shear modulus of the panel. Coppens [1] was one of the first using this method to test the elastic constants of PBs under the four sides completely free (FFFF) boundary condition by hanging them up on rubbers ties. A simultaneous measurement of elastic properties of a full-sized plywood panel with one edge simply supported and three edge free (SFFF) boundary condition by this vibration method was conducted by Sobue and Katoh [2]. The dynamic elastic properties of full-scale PB and MDF panels were tested using the same vibration technique in a vertical cantilever (CFFF) arrangement [3]. An on-line non-destructive apparatus, called VibraPann, was developed for evaluation of the *E* and strength in both major and minor axes for wood-based panels [5]. A laboratory testing apparatus was used to measure the dynamic *E* and dynamic viscoelasticity of full-sized WCPs supported on their two nodal lines by a vibration testing way [18,20]. Based on a modal testing technique, Zhou et al. [21] developed an NDE method for full-sized engineered wood-based panels with a boundary condition, in which two opposite sides were simply supported and the other two sides were free (SFSF). In terms of modal testing, the influence of the three boundary conditions (STSF, FFFF, and SFFF) for measurement of elastic constants of engineered wood-based panels was studied [19]. In addition, finite element (FE) modelling was also used for estimation of the elastic properties of full-sized WCPs combined with the NDE method [4,7,14]. These elastic constants were then estimated by minimizing the relative errors between experimental frequency and FE modelled values by an iteration process. Larsson [4] and Niederwestberg [14] tested full-sized oriented strand board (OSB) and cross-laminated timber (CLT) under the FFFF boundary condition using modal analysis, respectively. Gsell et al. [7] developed an automated procedure to determine global elastic properties of full-scale CLT through both experimental modal analysis and an analytical model based on Reddy’s higher order plate theory. All three shear moduli and the two in-plane MOE were identified by minimizing the difference between measured and estimated natural frequencies using the least square method. The conclusion reached in all of these works is that there is a good correlation between elastic constants of full-sized WCPs obtained by this panel-vibrational method and those measured by the traditional static method. However, due to poor operation, hitherto it seems the reported methods in these studies above are only applicable to conduct in the laboratory and not widely used for on-line property evaluation of full-sized WCPs. Another conclusion can be also drawn from these studies that the elastic properties of WCPs are closely associated with their vibration modal parameters. In order to determine elastic properties of full-sized WCP, the key is to determine the vibration mode shapes required and measure the corresponding natural frequencies of the panel under the given boundary condition.

In many cases, small MDF and PB specimens are always regarded as being in-plane quasi-isotropic materials, which have nearly the same property values in the major and minor directions [6,9,12,17,22]. However, full-sized MDF and PB panels can be modelled as orthotropic materials with non-uniform values in the major, minor, and thickness directions in many studies [3,15,19,21]. Zhang et al. [23] also found that *E* along the major direction was 56% higher than that along the minor direction for a 20 mm thick full-sized MDF panels using cantilever-beam bending. Thus, full-sized MDF and PB panels were considered to be orthotropic materials in this study.

The objective of this presented study was to lay a foundation for determining elastic properties of full-sized WCP by a vibration method for on-line non-destructive testing. The first nine modal parameters (natural frequency and mode shape) of the vibration of full-sized WCPs supported on four nodes were tested by experimental modal analysis. The panels tested include MDF and PB with three different thicknesses. Results from experimental modal testing were compared with theoretical modal analysis by COMSOL Multiphysics^®^ software (COMSOL Inc., Stockholm, Sweden). The sensitivity analysis was then performed to determine the sensitive modes for calculation of *E* in both major and minor axes, as well as the in-plane shear modulus of full-sized MDF and PB panels with four nodes of support. 

## 2. Relationship between Modal Parameters and Elastic Properties of Full-Sized WCPs

A full-sized WCP is assumed for an orthotropic thin-plate model in this study. For a thin rectangular orthotropic plate, neglecting the effects of shear deformation and rotatory inertia, its governing differential equation for the transverse vibration can be expressed as follows [24]:

(1) $$D_{x}\frac{\partial^{4}\omega }{\partial x^{4}}+D_{y}\frac{\partial^{4}\omega }{\partial y^{4}}+2\left(D_{1}+2D_{xy}\right)\frac{\partial^{4}\omega }{\partial x^{2}\partial y^{2}}+\rho h\frac{\partial^{2}\omega }{\partial t^{2}}=0$$

(2) $$D_{x}=\frac{E_{x}h^{3}}{12(1-\nu_{xy}\nu_{yx})}$$

(3) $$D_{y}=\frac{E_{y}h^{3}}{12(1-\nu_{xy}\nu_{yx})}$$

(4) $$D_{1}=D_{x}\nu_{yx}=D_{y}\nu_{xy}$$

(5) $$D_{xy}=\frac{G_{xy}h^{3}}{12}$$

where *D_x_* and *D_y_* are the flexural rigidities along the major and minor directions of the plate, *D*_1_ is the equivalent rigidity; *D_xy_* is the torsional rigidity; *ρ* is the mass density of the plate; *h* is the thickness of the plate; $\nu_{xy}$ and $\nu_{yx}$ are the Poisson’s ratios; and *E_x_*, *E_y_*, and *G_xy_* are the *E* along the major and minor directions and in-plane shear modulus, respectively.

**Figure 1:**
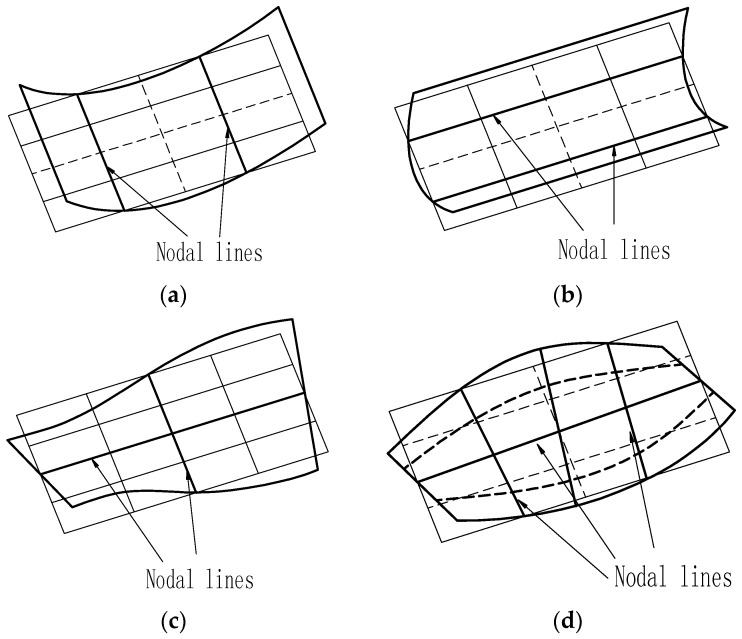
Four mode shapes. (**a**) Mode shape of mode (2, 0); (**b**) mode shape of mode (0, 2); (**c**) mode shape of mode (1, 1); and (**d**) mode shape of mode (2, 1).

In this study, the four-node support is chosen for modal testing and analysis of full-sized panels because vibration tests under such support conditions can be readily implemented for on-line non-destructive testing. The four-node support refers to a full-sized panel being supported on four node points, the intersections of two nodal lines of mode (2, 0) and mode (0, 2). These four nodal lines are located at 22.4% and 77.6% of its length and width, respectively. This support can be regarded as a special FFFF boundary condition, therefore, the solution of Equation (1) is based on the Rayleigh method with one-term deformation expression for the specific modes presented by Hearmon [25]. The resonant frequencies for an orthotropic plate can be expressed as [5,19,26]:

(6) $$f_{\left(m,n\right)}=\frac{1}{2\pi }\sqrt{\frac{1}{\rho h}}\sqrt{D_{x}\frac{\alpha_{1}}{a^{4}}+D_{y}\frac{\alpha_{2}}{b^{4}}+2D_{1}\frac{\alpha_{3}}{a^{2}b^{2}}+4D_{xy}\frac{\alpha_{4}}{a^{2}b^{2}}}$$

where *f*_(*m*, *n*)_ is the natural frequency of a specific mode (*m*, *n*); *a* and *b* are the length and width of the plate; (*m*, *n*) identifies the mode, where *m* and *n* represent the number of node lines including the simply supported sides in the minor and major directions of the plate, respectively; and *α*_1(*m*, *n*)_, α_2(*m*, *n*)_, α_3(*m*, *n*)_, and α_4(*m*, *n*)_ are the coefficients for each specific mode related to the boundary conditions. Four mode shapes are presented in Figure 1 and the corresponding coefficients *α_k_* are given in Table 1 [5,27].

Substituting Equations (2)–(5) into Equation (6), and considering the corresponding coefficients for four mode shapes shown in Table 1, the elastic properties *E_x_*, *E_y_*, and *G_xy_* of a full-sized WCP can be derived as follows [19]:

(7) $$E_{x}=\frac{48\pi^{2}\rho a^{4}(1-\nu_{xy}\nu_{yx})}{500.6h^{2}}{f_{\left(2,0\right)}}^{2}$$

(8) $$E_{y}=\frac{48\pi^{2}\rho b^{4}(1-\nu_{xy}\nu_{yx})}{500.6h^{2}}{f_{\left(0,2\right)}}^{2}$$

(9) $$G_{xy}=\frac{\pi^{2}\rho a^{2}b^{2}}{12h^{2}}{f_{\left(1,1\right)}}^{2}$$

(10) $$G_{xy}=\frac{12a^{2}}{593.76}\left(\frac{f_{\left(2,1\right)}^{2}\pi^{2}\rho b^{2}}{h^{2}}-\frac{500.6E_{x}b^{2}}{48a^{4}(1-\nu_{xy}\nu_{yx})}\right)$$

where $\nu_{xy}\nu_{yx}$ is substituted with 0.01 for most wood materials [28].

When the panel geometry size (*a*, *b*, *h*) and density (*ρ*) are given, the relationship between modal parameters and elastic properties of full-sized WCPs can be obtained by inputting these parameters into Equations (7)–(10).

## 3. Materials and Methods 

### 3.1. Materials

Two types of WCPs were used in this study, including MDF and PB panels. Three full-sized MDF panels and three full-sized PB panels were provided by two different manufacturers. These panels were made of mixed softwood and hardwood species using urea-formaldehyde resin according to the communication with the manufactures. Both MDF and PB panels had three different nominal thicknesses, 12, 15, and 18 mm. The average moisture content of the MDF and PB panels were about 4% and 5%. The specifications of the samples are presented in Table 2. 

### 3.2. Experimental Modal Analysis

Experimental modal analysis is a technique for determining the modal parameters (modal or natural frequency and mode shape) of a vibrating structure [29]. The frequency response function (FRF) is generated by the relationship of the structure’s dynamic response and the excitation causing it. Modal testing of the panels were conducted in a room with a relative humidity of 30 ± 5% and a temperature of 20 ± 2 °C. To obtain the mode shapes, a response (or excitation) reference point was set and the FRFs were measured at various points distributed on a preset grid. The mode shapes can then be reconstructed by means of the signs and magnitudes of the imaginary part of each measured natural frequency.

**Figure 2:**
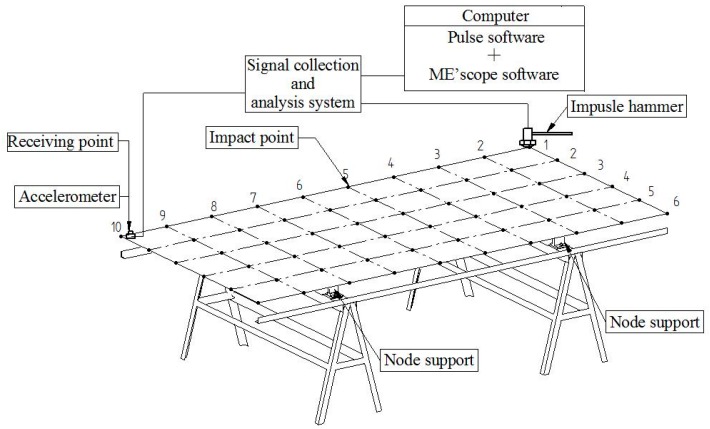
System diagram of modal testing and modal analysis for full-sized WCPs.

Figure 2 shows the system diagram of modal testing and modal analysis for full-sized WCPs. The modal testing was performed using a Pulse signal collection and analysis system (type 3560-C, Brüel and Kjær, Copenhagen, Denmark). The vibrational response of the full-sized WCP tested was measured by an accelerometer (type 4507-B-004, Brüel and Kjær, Copenhagen, Denmark), following the impact by an impulse hammer (model 2302-10, Brüel and Kjær, Copenhagen, Denmark). The signals were acquired and digitized via the chassis of this system. The modal parameters of the panels were obtained by some modal parameter identification methods using the post-processing software of this system. 

The supporting system designed for vibration testing of the panels is a four-node support as shown in Figure 2. In view of the reciprocity hypothesis satisfied between the excitation and response of the system, a single-point receiver coupled with multi-point excitation was adopted for the modal testing. For each panel under the four-node support, the receiver sensor is attached to the panel at a fixed position (plate corner), the panel is excited using an impulse hammer at a series of locations as specified by the grid points (Figure 2). In order to obtain ideal modal shapes, a total of 60 measurement points were selected and marked on each panel (5 × 9 grids). 

During the modal testing, a full-sized panel was symmetrically placed on the four-node support test bench. All of the measurement points on the panel were tapped successively to obtain the FRFs. To ensure the measurement accuracy and reduce random error, each measurement point was tested three times to obtain the average value of the FRFs. Then all the FRFs tested were imported into ME’ scope software (Vibrant Technology Inc., Scotts Valley, CA, USA) to carry through the modal parameter identification and mode shape simulation. The natural frequencies and mode shapes of the first nine vibration modes of the panel were obtained in this software.

### 3.3. Theoretical Modal Analysis

To further verify the modal parameters of the full-sized WCPs measured through the experimental modal testing, a theoretical modal analysis was conducted for each panel to obtain calculated frequencies and mode shapes of the first nine modes. Theoretical modal analysis is a modeling process that uses the FE method to discretize a vibrating structure, build a mathematical model about system eigenvalues, and solve the system eigenvalues and eigenvectors, namely modal frequencies and mode shapes of the structure [30]. In this study, the COMSOL Multiphysics^®^ software was used for theoretical modal analysis of the panels. Full-sized WCPs were modelled as a structural plate using plate element of the mixed interpolation of tensorial component (MITC) type, which can be used for analyzing both thin and thick plates. In this model, the major direction of the panel was set as *X* axis, the minor direction as *Y* axis and the thickness direction as *Z* axis. Since full-sized WCPs were regarded as orthotropic materials, the model for each panel was established based on the input parameters including two *E* (*E_x_* and *E_y_*), three shear modulus (*G_xy_*, *G_yz_*, *G_xz_*), one Poisson’s ratio ($\nu_{xy}$), density (*ρ*), and geometry size (*a*, *b*, *h*). Among them, *E_x_* and *E_y_* can be initially estimated based on the natural frequencies of modes (2, 0) and (0, 2) obtained in the above experimental modal testing according to Equations (7) and (8), respectively. The in-plane shear modulus *G_xy_* was initially estimated using the natural frequency of mode (1, 1) obtained in the experimental modal testing according to Equation (9). Out-of-plane shear moduli *G_yz_* and *G_xz_* were determined by conducting three-point bending test on small MDF and PB specimens cut from the full-sized panels at different short spans [31,32]. Previous studies showed that the Poisson’s ratio of a panel had a very small influence on the natural frequencies [3,4,26]. Hence, the value of the Poisson ratio from the literature data was used in this study [33,34]. In addition, full-sized WCPs were considered as thin plates in this software and thus were modelled using four elastic parameters (*E_x_*, *E_y_*, *G_xy_* and $\nu_{xy}$).

In addition of density and geometry size (Table 2), other input parameters are shown in Table 3. Grid division methods had a very small effect on the result of the FE analysis for the plate model, and the model was conveniently meshed using free triangular meshing in the software. In order to ensure the numerical accuracy of the FE analysis, an initial and a smaller element mesh were used to obtain calculated frequencies, respectively. Through convergence analysis between calculated frequencies, a fit element mesh was chosen with the modal meshed with the largest element of 24.4 mm and the smallest element of 0.005 mm. According to the supporting system of the panel, the degree of freedom of four nodes on the Z axis was restricted in the model.

Since there were no closed-form solutions for the rectangular plate with FFFF direction condition, and the Rayleigh method applied for the calculation of Equation (1) was an approximate numerical method, the estimation of *E_x_*, *E_y_*, and *G_xy_*, as three main input parameters, need to be refined. Those initially-estimated three elastic constants were updated according to the following equations [4]:

(11) $$\frac{\left|f_{\left(2,0\right)}^{\mathrm{FE}}-f_{\left(2,0\right)}^{\mathrm{EM}}\right|}{f_{\left(2,0\right)}^{\mathrm{EM}}}>0.01\Rightarrow E_{x}(N+1)=E_{x}(N){\left(\frac{f_{\left(2,0\right)}^{\mathrm{EM}}}{f_{\left(2,0\right)}^{\mathrm{FE}}}\right)}^{2}$$

(12) $$\frac{\left|f_{\left(0,2\right)}^{\mathrm{FE}}-f_{\left(0,2\right)}^{\mathrm{EM}}\right|}{f_{\left(0,2\right)}^{\mathrm{EM}}}>0.01\Rightarrow E_{y}(N+1)=E_{y}(N){\left(\frac{f_{\left(0,2\right)}^{\mathrm{EM}}}{f_{\left(0,2\right)}^{\mathrm{FE}}}\right)}^{2}$$

(13) $$\frac{\left|f_{\left(1,1\right)}^{\mathrm{FE}}-f_{\left(1,1\right)}^{\mathrm{EM}}\right|}{f_{\left(1,1\right)}^{\mathrm{EM}}}>0.01\Rightarrow G_{xy}(N+1)=G_{xy}(N){\left(\frac{f_{\left(1,1\right)}^{\mathrm{EM}}}{f_{\left(1,1\right)}^{\mathrm{FE}}}\right)}^{2}$$

where *N* is number of iterations; *f*_FE_ represents the calculated frequency obtained by the FE method; and *f*_EM_ represents the experimental frequency obtained in the experimental modal testing. The experimental frequencies for modes (2, 0), (1, 1), and (0, 2) were compared with the corresponding initial calculated frequencies (Table 4). The initial estimates of *E_x_* (1), *E_y_* (1), and *G_xy_* (1) were updated according to Equations (11)–(13), respectively. The updated *E_x_* (2), *E_y_* (2), and *G_xy_* (2) were then used as new input parameters for another FE analysis. New frequencies were calculated next and were compared with the corresponding experimental ones again. Iteration step number one was then implemented. It was found that it was often effective to have three iterations.

### 3.4. Sensitivity Analysis

Theoretically, any three natural frequencies can be used to calculate the three elastic constants (*E_x_*, *E_y_*, and *G_xy_*) of the panel under a FFFF boundary condition according to Equation (6). For a specific boundary condition, however, some modes may be more sensitive to the variation of some elastic constants than others; therefore, the identification of the most sensitive modes for calculation was needed. One approach of sensitivity analysis is to change 10% of each elastic constant in a FE model and check the relative frequency differences [4,35]. Based on this approach, the sensitivity (Δ) can be defined as the frequency change as a specific elastic constant change:

(14) $$\Delta =\frac{|f_{\left(m,n\right)}\left[\mathrm{inc}\right]-f_{\left(m,n\right)}\left[\mathrm{ref}\right]|}{f_{\left(m,n\right)}\left[\mathrm{ref}\right]}\times 100\%$$

where *f*_(*m*,*n*)_[inc] represents the calculated frequency of mode (*m*, *n*) when the input specific elastic constant in the FE model increases 10%, and *f*_(*m*,*n*)_[ref] represents the reference frequency. After three iterations in the above theoretical modal analysis, the calculated frequencies obtained were used as reference frequency values. Likewise, these input elastic constants for calculating reference frequencies were chosen as reference values of specific elastic constants, shown in Table 5. At first, the constant *E_x_* was increased by 10% from its reference value and a new FM calculation was performed. Relative deviations between *f*_(*m*,*n*)_[inc] and *f*_(*m*,*n*)_[ref] of the first nine modes of vibration were calculated. Similar calculations were performed for *E_y_* and *G_xy_*. Scatterplots were used to indicate results of sensitivity analysis for full-sized WCPs tested.

## 4. Results and Discussion

### 4.1. Comparison between Mode Shapes of Full-Sized WCPs

**Figure 3:**
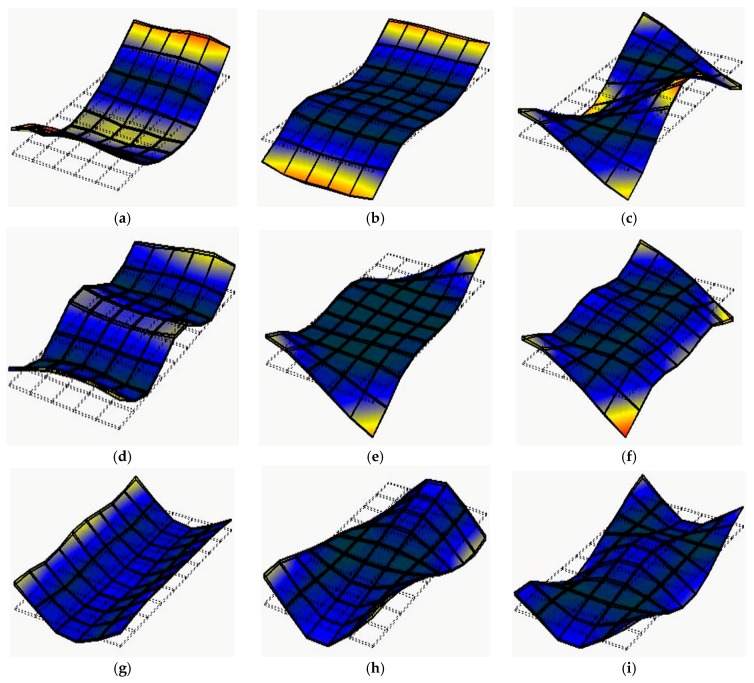
The first nine mode shapes of MDF12 obtained through experimental modal analysis. (**a**) Mode shape of the first mode (2, 0); (**b**) mode shape of the second mode (3, 0); (**c**) mode shape of the third mode (2, 1); (**d**) mode shape of the fourth mode (4, 0); (**e**) mode shape of the fifth mode (1, 1); (**f**) mode shape of the sixth mode (4, 1); (**g**) mode shape of the seventh mode (0, 2); (**h**) mode shape of the eighth mode (1, 2); and (**i**) mode shape of the ninth mode (2, 2).

**Figure 4:**
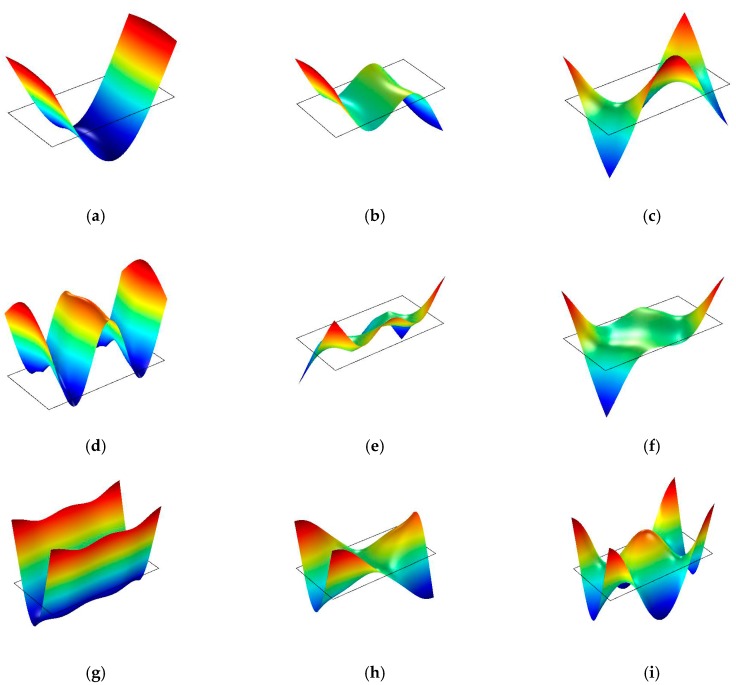
The first nine mode shapes of MDF12 obtained through theoretical modal analysis. (**a**) Mode shape of the first mode (2, 0); (**b**) mode shape of the second mode (3, 0); (**c**) mode shape of the third mode (2, 1); (**d**) mode shape of the fourth mode (4, 0); (**e**) mode shape of the fifth mode (1, 1); (**f**) mode shape of the sixth mode (4, 1); (**g**) mode shape of the seventh mode (0, 2); (**h**) mode shape of the eighth mode (1, 2); and (**i**) mode shape of the ninth mode (2, 2).

The first nine mode shapes of MDF12 obtained from the experimental and theoretical modal analysis are shown in Figure 3 and Figure 4, respectively. It can be seen from these figures that the same order for MDF12 shows the same mode shape obtained in the experimental and theoretical modal analysis: the mode shape at the first, second, and fourth order is flexural along the length direction; the mode shape at the third and sixth order is flexural along the length direction combined with torsion; the mode shapes at the seventh, eighth, and ninth order are flexural along the width direction, flexural along the width direction combined with torsion, and flexural along the length direction combined with flexural along the width direction, respectively; it is noted that the mode shape at the fifth order is made up of torsion consisted of the left and right part of two nodal lines along the width direction and nearly static part between two nodal lines along the width direction, thus the mode shape at the fifth order can be considered as torsion in mode (1, 1) neglecting the static part between two nodal lines along the width direction. In addition, the first nine order mode shapes for MDF15, MDF18, and PB with three thicknesses obey the same rule in the experimental and theoretical modal analysis. For the first nine mode shapes of both full-sized MDF and PB panels of three thicknesses, the same order shows the same mode shape in the experimental and theoretical modal analysis, which verifies the feasibility of these two modal analyses. 

### 4.2. Comparison between Natural Frequencies of Full-Sized WCPs

The first nine natural frequencies of full-sized MDF and PB panels with three different thicknesses in the experimental and theoretical modal analysis are given in Table 6 and Table 7, respectively. The calculated frequencies are the values obtained after three iterations in the theoretical modal analysis. The relative deviation (Diff) between the experimental frequency and calculated frequency are also given in Table 6 and Table 7. It can be seen from the table that the experimental frequencies at the first nine modes are in good agreement with the calculated frequencies and the Diffs between all the frequencies are within 6% for all of the panels; the Diffs between the frequencies at the first, fifth and seventh order are within 1% after three iterations.

**Figure 5:**
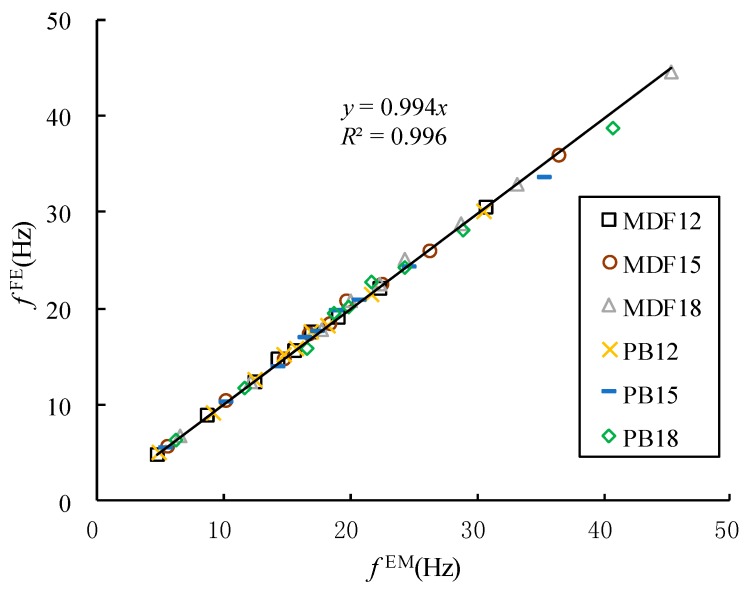
The relationship between the experimental frequencies and calculated frequencies of the full-sized MDF and PB panels in three different thicknesses.

Figure 5 shows the relationship between the experimental frequencies and calculated frequencies in the first nine modes for the full-sized MDF and PB panels. There is a strong linear relationship between the experimental frequencies and calculated frequencies of all of the panels. This indicates that the vibration modal parameters of full-sized WCPs obtained through experimental modal analysis are valid. 

### 4.3. Sensitivity Analysis for Modal Parameters

**Figure 6:**
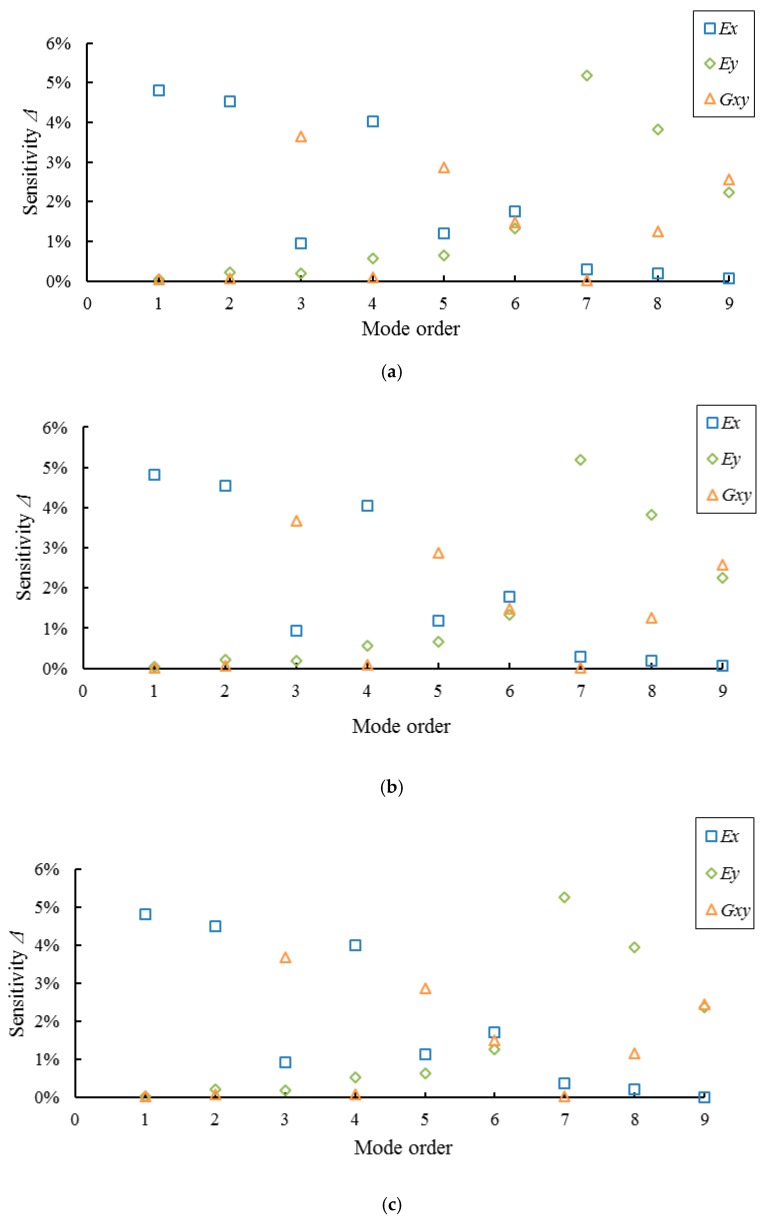
Results of sensitivity analysis for full-sized MDF panels with three different thicknesses. (**a**) MDF12; (**b**) MDF15; and (**c**) MDF18.

**Figure 7:**
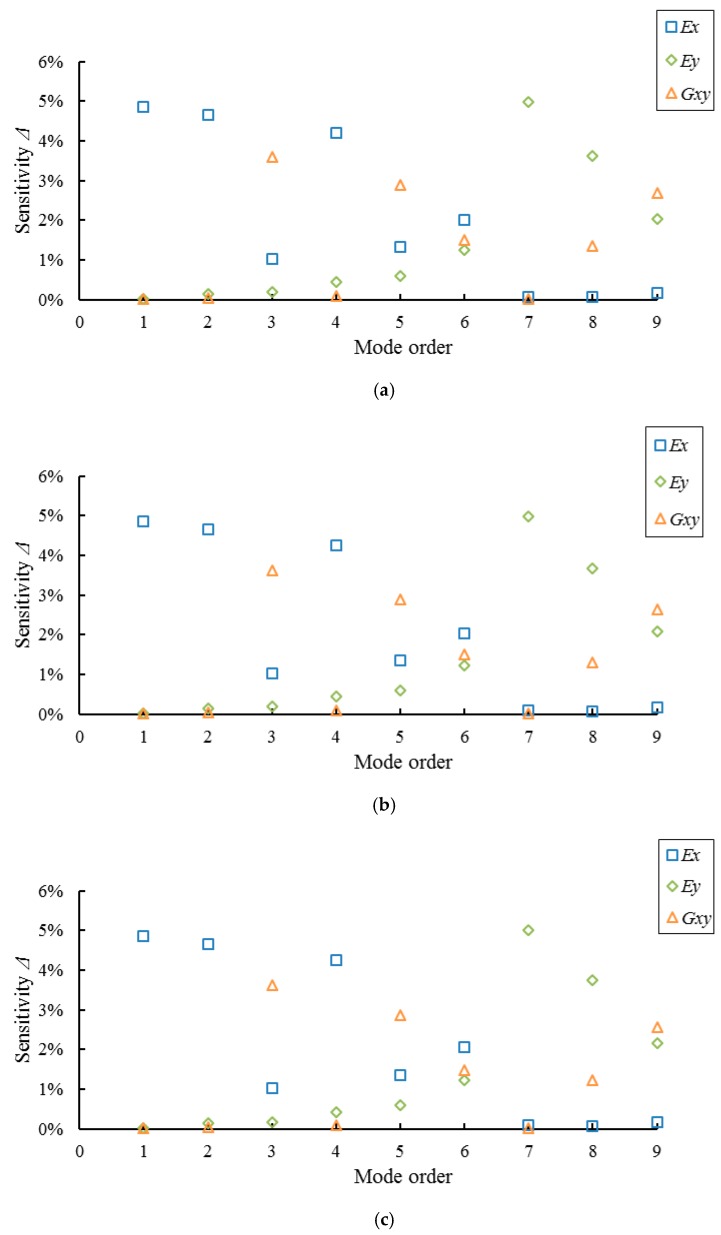
Results of sensitivity analysis for full-sized PB panels with three different thicknesses. (**a**) PB12; (**b**) PB15; and (**c**) PB18.

Results of sensitivity analysis for the modal parameters of full-sized MDF and PB panels are shown in Figure 6 and Figure 7, respectively. It can be seen from the figures that the frequencies of the first, second, and fourth orders, namely modes (2, 0), (3, 0), and (4, 0), are most sensitive to the change in *E_x_*; frequencies of the third, fifth and ninth orders, namely modes (2, 1), (1, 1), and (2, 2), are most sensitive to the change in *G_xy_*; frequencies of the seventh and eighth orders, namely modes (0, 2) and (1, 2), are most sensitive to the change in *E_y_*; frequency of the sixth order mode (4, 1) has nearly the same sensitivities to the changes in *E_x_*, *E_y_*, and *G_xy_*. The results also indicate that the full-sized MDF and PB panels with different thicknesses show the same results, indicating that the panel thickness has no effect on the sensitivities of the mode’s frequency with respect to changes in *E_x_*, *E_y_*, and *G_xy_*.

In addition, the boundary condition of a full-sized WCP under the four-node support can be considered completely free on four sides. However, in fact, the six vibration modes (2,0), (2,1), (1,1), (0, 2), (1,2), and (2, 2) of the full-sized WCPs tested remain free when the other three modes (3, 0), (4, 0), and (4, 1) are attenuated (the four supports are not placed on the nodes for these three modes [36]). Therefore, the corresponding frequencies of modes (3, 0) (4, 0), and (4, 1) are not suitable for calculating the elastic constants of full-sized MDF and PB panels supported on four nodes according to Equation (6). In summary, the sensitive modes for calculating *E_x_* and *E_y_* of full-sized WCPs are (2, 0) and (0 or 1, 2), and the sensitive modes for calculating *G_xy_* are (2, 1), (1, 1), and (2, 2). In view of the strongest sensitivity and the convenience of the frequency testing, the frequencies of modes (2, 0), (0, 2), and (2, 1) are preferred in calculating *E_x_*, *E_y_*, and *G_xy_* of full-sized WCPs based on Equations (7), (8), and (10), respectively.

## 5. Conclusions

In this study, the natural frequencies and mode shapes of the first nine vibration modes for full-sized MDF and PB panels supported on four nodes were determined using experimental modal analysis. Modal parameters of the panels obtained from experimental modal testing were compared with the results of theoretical modal analysis. Sensitivity analysis was performed to identify the vibration modes that are most sensitive to the changes in elastic constants. Based on the results and analysis, we concluded the following:

(1) Mode shapes of the full-sized MDF and PB panels obtained from modal testing are in good agreement with those obtained from theoretical modal analyses. A strong linear relationship exists between the measured natural frequencies and the calculated frequencies of the panels.
(2) The frequencies of modes (2, 0), (0, 2), and (2, 1) of full-sized MDF and PB panels under the four-node support condition are identified as the characteristic frequencies for determining moduli of elasticity in major and minor directions and the in-plane shear modulus of the panels. Panel thickness has no effect on the sensitivity of the frequencies in terms of elastic property prediction.
(3) The results of this study indicate that a free vibration system under four-node support has a good potential to be used for evaluating the elastic properties of full-sized WCPs.

Future research is planned to apply this technique to full-sized commercial WCPs in a large sample size and test the feasibility of using modal parameters to predict both elastic constants and other performance characteristics of full-sized panels.

## Figures and Tables

**Table 1:** Corresponding coefficients for four mode shapes.

Mode		*α*_1_	*α*_2_	*α*_3_	*α*_4_
*m*	*n*				
2	0	500.6	0	0	0
0	2	0	500.6	0	0
1	1	0	0	0	144
2	1	500.6	0	0	593.76

**Table 2:** Specifications of full-sized WCPs tested.

Panel Code	Panel Sizes (Thickness × Width × Length, mm)	Average Density (kg/m^3^)
MDF12	12.0 × 1220 × 2440	803
MDF15	15.0 × 1220 × 2440	723
MDF18	18.2 × 1220 × 2440	705
PB12	11.9 × 1220 × 2440	667
PB15	15.3 × 1223 × 2445	687
PB18	18.2 × 1224 × 2445	705

**Table 3:** Input parameters of the full-sized WCPs used in theoretical modal analysis.

Panel Code	MOE (GPa)		Shear Modulus (GPa)			Poisson’s Ratio
	*E_x_*	*E_y_*	*G_xy_*	*G_yz_*	*G_xz_*	*ν_xy_*
MDF12	4.09	4.22	1.99	0.12	0.13	0.3
MDF15	3.28	3.39	1.60	0.16	0.21	
MDF18	3.10	3.66	1.55	0.17	0.17	
PB12	3.82	3.24	1.70	0.29	0.29	0.2
PB15	2.93	2.65	1.32	0.40	0.42	
PB18	2.82	2.64	1.25	0.48	0.50	

**Table 4:** Initial calculated frequencies of full-sized WCPs in theoretical modal analysis.

Panel Code	Initial Calculated Frequencies (Hz)								
	1	2	3	4	5	6	7	8	9
MDF12	4.70	8.85	13.67	14.77	16.98	18.43	19.83	23.53	33.09
MDF15	5.53	10.42	16.14	17.44	20.08	21.83	23.37	27.76	39.09
MDF18	6.63	12.48	19.48	20.86	24.22	26.33	30.02	34.97	48.23
PB12	4.92	9.17	13.82	15.26	17.09	18.44	18.41	22.51	32.43
PB15	5.43	10.13	15.33	16.90	19.00	20.58	20.95	25.41	36.31
PB18	6.27	11.70	17.57	19.51	21.82	23.74	24.60	29.58	41.92

**Table 5:** Reference values of moduli of elasticity *E_x_*, *E_y_*, and in-plane shear modulus *G_xy_*.

Panel Code	Elastic Constants (GPa)		
	*E_x_*	*E_y_*	*G_xy_*
MDF12	4.09	3.92	1.55
MDF15	3.28	3.14	1.24
MDF18	3.10	3.37	1.22
PB12	3.82	3.17	1.31
PB15	2.93	2.58	1.01
PB18	2.82	2.58	0.94

**Table 6:** The first nine natural frequencies of the full-sized MDF panels obtained through the experimental and theoretical modal analysis ^a^.

Order	Panel Code								
	MDF12			MDF15			MDF18		
	EF (Hz)	CF (Hz)	Diff (%)	EF (Hz)	CF (Hz)	Diff (%)	EF (Hz)	CF (Hz)	Diff (%)
1	4.70	4.70	0.05	5.53	5.53	0.04	6.62	6.62	0.01
2	8.67	8.81	1.64	10.20	10.38	1.77	12.20	12.43	1.85
3	12.50	12.42	0.66	14.80	14.62	1.23	17.70	17.75	0.26
4	14.20	14.67	3.31	16.70	17.32	3.69	20.10	20.72	3.08
5	15.60	15.67	0.47	18.40	18.49	0.47	22.30	22.40	0.47
6	16.90	17.52	3.66	19.70	20.72	5.16	24.30	25.05	3.07
7	19.10	19.05	0.28	22.50	22.44	0.28	28.80	28.70	0.34
8	22.30	22.11	0.84	26.20	26.05	0.57	33.20	32.86	1.03
9	30.70	30.45	0.81	36.40	35.88	1.43	45.30	44.48	1.81

^a^ EF—experimental frequencies; CF—calculated frequencies.

**Table 7:** The first nine natural frequencies of the full-sized PB panels obtained through the experimental and theoretical modal analysis ^a^.

Order	Panel Code								
	PB12			PB15			PB18		
	EF (Hz)	CF (Hz)	Diff (%)	EF (Hz)	CF (Hz)	Diff (%)	EF (Hz)	CF (Hz)	Diff (%)
1	4.94	4.92	0.34	5.45	5.43	0.33	6.29	6.27	0.32
2	9.15	9.16	0.10	10.20	10.12	0.80	11.60	11.68	0.67
3	12.50	12.55	0.38	14.30	13.87	3.03	16.50	15.81	4.20
4	14.70	15.21	3.45	16.35	16.84	3.00	18.70	19.44	3.98
5	15.70	15.78	0.52	17.40	17.49	0.54	19.90	20.02	0.58
6	16.90	17.63	4.30	18.90	19.63	3.86	21.60	22.61	4.66
7	18.20	18.19	0.03	20.70	20.69	0.03	24.30	24.29	0.04
8	21.60	21.50	0.46	24.70	24.26	1.78	28.90	28.22	2.34
9	30.50	30.01	1.61	35.30	33.54	4.98	40.80	38.62	5.34

^a^ EF—experimental frequencies; CF—calculated frequencies.
